# The constitutive activity of the viral-encoded G protein-coupled receptor US28 supports a complex signalling network contributing to cancer development

**DOI:** 10.1042/BST20190988

**Published:** 2020-08-11

**Authors:** Carole A. Daly, Martine J. Smit, Bianca Plouffe

**Affiliations:** 1 Wellcome-Wolfson Institute for Experimental Medicine, Queen's University Belfast, Belfast, U.K.; 2 Amsterdam Institute for Molecular and Life Sciences, VU University Amsterdam, Amsterdam, The Netherlands

**Keywords:** cancer, chemokine receptor, constitutive activity, signalling network, US28, viral GCPR

## Abstract

US28 is a viral G protein-coupled receptor (GPCR) encoded by the human cytomegalovirus (HCMV). This receptor, expressed both during lytic replication and viral latency, is required for latency. US28 is binding to a wide variety of chemokines but also exhibits a particularly high constitutive activity robustly modulating a wide network of cellular pathways altering the host cell environment to benefit HCMV infection. Several studies suggest that US28-mediated signalling may contribute to cancer progression. In this review, we discuss the unique structural characteristics that US28 acquired through evolution that confer a robust constitutive activity to this viral receptor. We also describe the wide downstream signalling network activated by this constitutive activation of US28 and discuss how these signalling pathways may promote and support important cellular aspects of cancer.

## Introduction

G protein-coupled receptors (GPCRs) represent the largest transmembrane receptor family, including ∼800 members and are target of over 30% of all therapeutic drugs [1] due to their substantial involvement in physiological processes. GPCRs modulate these processes by regulating intracellular signalling cascades in response to neurotransmitters, photons, ions, odorants and hormones. Agonist binding to these receptors at the plasma membrane stabilise an active GPCR conformation detected by heterotrimeric G proteins composed of an α, β and γ subunit [2]. This leads to the exchange of GDP for GTP from the Gα subunit triggering its dissociation from the Gβγ dimer. Both the Gα and the Gβγ subunits can modulate various cell signalling pathways by activating downstream effectors [3].

There are four families of heterotrimeric Gα proteins, based on the sequence and functional similarities. The Gαs/olf family is composed of two members: Gαs and Gαolf, both activating their effector adenylyl cyclase (AC), responsible for the conversion of adenosine triphosphate (ATP) into cyclic adenosine monophosphate (cAMP). The Gαi/o family is composed of six members (Gαi1, Gαi2, Gαi3, GαoA, GαoB and Gαz) inhibiting AC and consequently cAMP production. The third family (Gαq/11) is composed of four members: Gαq, Gα11, Gα14 and Gα16. These Gα proteins activate phospholipase Cβ (PLCβ), which cleaves phosphatidylinositol-4,5-bisphosphate (PIP_2_) into diacylglycerol (DAG) and inositol trisphosphate (IP_3_). DAG activates protein kinase C (PKC) and IP_3_ opens the calcium channel IP_3_ receptor on the endoplasmic reticulum (ER) membrane to release calcium from the ER into the cytosol. The increased concentration of cytosolic calcium by IP_3_ leads to activation of calcium-dependent proteins, such as PKC and calmodulin. Members from the Gαq/11 family are also reported to activate the Ras homolog family member A (RhoA), leading to activation of the Rho-associated protein kinase (ROCK) involved in the actin cytoskeletal reorganisation [4,5]. The last Gα protein family (Gα12/13) is composed of two isoforms: Gα12 and Gα13. These Gα proteins generally activate the Rho/ROCK pathway. Gβγ can also modulate the activity of downstream effectors such as AC, PLCβ, voltage-gated calcium channels and inwardly rectifying potassium channels [6]. Although, due to the relatively higher amounts of Gα proteins from the Gαi/o family, activation of these Gα proteins is thought to be the primary source of Gβγ-mediated signalling.

G protein dissociation and signalling is followed by the recruitment of kinases, such as the G protein-coupled receptor kinases (GRKs) to the receptor [7] and phosphorylation of the GPCR at specific serine or threonine residues in the intracellular loops and C-terminal tail (CT), producing a distinct pattern of phosphorylation, or ‘barcode' [8]. A protein called β-arrestin is then recruited to the phosphorylated receptor resulting in GPCR uncoupling from G proteins but also recruits adaptor proteins required for receptor internalisation via clathrin-coated pits, promoting GPCR internalisation into early endosomes. The ‘barcode' dictates the fate of the receptor. While some GPCRs are targeted to lysosomes for degradation, others are recycled back to the plasma membrane. Alternatively, other receptors undergo persistent G protein activation in early endosomes, while others are transported to the Golgi to activate G proteins from this cellular compartment [9]. β-arrestin has been shown to be required for G protein signalling in intracellular compartments by forming a transient complex together with the receptor and G proteins, called ‘megaplex' [10]. This megaplex has been hypothesised to occur for GPCRs having high affinity for β-arrestins.

Although the majority of GPCRs regulating human physiology are the product of a portion of the genome of an individual, a few GPCRs detected in a human body have a viral origin, acquired upon infection of the pathogen. One of these viruses is the human cytomegalovirus (HCMV), a member of the β-herpesviruses. Herpesviruses such as HCMV have evolved to encode viral GPCRs which are chemokine receptors believed to have been ‘hijacked' from host genomes during evolution and severely modified and optimised for the virus's own benefit [11]. These viral GPCRs often exhibit particular characteristics such as high constitutive activity or promiscuous binding of ligands. The HCMV encodes four GPCR homologues: UL33, UL78, US27 and US28. UL33, UL78 and US27 are orphan viral GPCRs with no known ligands, but US28 binds with high affinity to many CC-type chemokines as well as CX3CL1 (fractalkine) [12]. US28 has 38% sequence homology with the human CX3CR1 receptor [13]. A recent study demonstrated that US28 can accommodate at least thousands of engineered chemokines [14]. This ability of US28 to bind to a high number of chemokines has been proposed as an evolutionary mechanism to serve as a chemokine sink to subvert and escape the host's immune system [15]. Indeed, the CT of US28 is rich in serine and threonine residues which, once phosphorylated by GRKs or other protein kinases, lead to rapid internalisation [16]. This robust internalisation of US28 results in 80% of the receptors located intracellularly as found for other HCMV-encoded GPCRs, but not all other viral GPCRs which are found at the plasma membrane [17,18]. As chemokines attract immunomodulatory cells, the ability of US28 to bind and sequester a large range of chemokines inside the host's cells by rapid internalisation is beneficial for immune evasion [19].

## The molecular determinants of the constitutive activation of G proteins by US28

Despite the extraordinary ability to bind with high affinity to an impressive number of chemokines, US28 can also activate G proteins in absence of agonist through its robust constitutive activity. US28 has been reported to activate in a constitutive manner three families of Gα proteins: the Gαq/11 [17,20–31], Gα12/13 [28,32] and Gαi/o [31] families in several cell types. Ligand-induced US28 signalling has been also reported, and is associated to many important functional consequences of HCMV [14,20,32–37]. In studies related to US28 constitutive activity, the Gαq G protein isoform is the most studied. The high constitutive activation of Gαq by US28 leads to a potent activation of PLCβ [20,22,30,34,38]. It has been shown that inhibition of HCMV viral replication by the viral polymerase inhibitor phosphonoacetic acid (PAA) in HCMV-infected glioblastoma U373MG cells does not succeed to abrogate PLCβ activation, despite the fact that US28 protein expression was undetectable by western blot [20]. Although activation of the pathways downstream of PLCβ were not monitored, these results highlight the robust ability of US28 to activate Gαq in a constitutively active manner.

**Figure 1.**
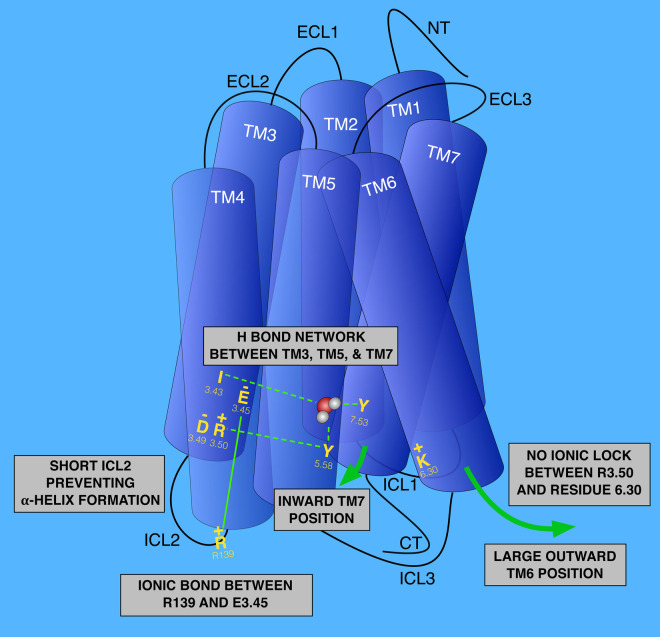
The main structural determinants responsible for the high constitutive activity of US28. The large outward position of TM6 due to the absence of ionic lock between residues 3.50 and 6.30 maintains US28 in an active conformation. The hydrogen bond network between TM3, TM5, and TM7 involving Y^7.53^, Y^5.58^, I^3.43^ and R^3.50^ induces an inward position of TM7. This inward position of TM7 is common in active GPCR structures. The ionic interaction between R139 in ICL2 and E^3.45^ instead of D^3.49^ destabilises the inactive state of US28. The shorter ICL2 preventing α-helix formation in this region also contributes to the absence of interaction between R139 and D^3.49^.

Recent crystal structures of US28 in complex with the human CX3CL1 reveal that US28 has evolved a highly stable active state [39]. Many structural features of US28 explain this constitutive activity. The conformation of the transmembrane (TM) 6 (TM6) in US28 is typical of an active-state GPCR [40]. Similarly to many active-state GPCRs, US28 exhibits a large outward movement at the intracellular end of TM6 [39] (Figure 1). This open position of TM6 is partly due to the absence of a conserved acidic residue at position 6.30 normally forming a salt bridge with the positively charged and conserved arginine residue at position 3.50 within the DRY motif [40,41] (Figure 1). The absence of such salt bridge, known as ‘ionic lock', prevents stabilisation of an inactive GPCR conformation. Another structural particularity of US28 contributing to its high constitutive activity is the presence of a hydrogen bond network connecting TM3, TM5 and TM7 present in previously solved active-state GPCR structures [40,42] (Figure 1). There is an inward position of TM7 caused by the formation of hydrogen bonds between tyrosine at position 7.53 with tyrosine 5.58 and isoleucine 3.43 through a water molecule [39] (Figure 1). The tyrosine 5.58, in turn, forms a hydrogen bound with the arginine at position 3.50 of the DRY motif (Figure 1). Finally, the structural environment around the aspartate at position 3.49, near the cytoplasmic end of TM3, also represents an adaptive evolutionary mechanism of US28 to promote a high constitutive activity. The arginine 139 in the second intracellular loop (ICL2) of US28 forms an ionic interaction with a unique glutamate at position 3.45 of US28 instead of contacting aspartate 3.49 [39] (Figure 1). This interaction potentially destabilises the inactive state of US28. ICL2 of US28 is also shorter by four residues in US28 compared with most class A GPCRs (Figure 1). This shorter ICL2 is believed to prevent the formation of an α-helix [39]. As this α-helix is known to help to position the arginine of ICL2 in a way to interact with the aspartate 3.49, the lack of ICL2 secondary structure may provide the required flexibility for the arginine in ICL2 to adopt different structural states.

Although the DRY motif is not forming an ‘ionic lock' with an acidic residue in TM6, it interacts with Gα proteins and is consequently responsible for the observed constitutive activity of US28. Changes within the DRY motif by a point mutation in the arginine residue 3.50 (US28-R129A) disrupts and uncouples G protein binding to the receptor and markedly attenuates signalling [43]. As this G protein-uncoupled receptor is nonetheless expressed at similar levels than wild-type US28 [43,44], it was used in many studies [20,24,26,27,38] to demonstrate the importance of US28-mediated constitutive activation of G proteins in engaging several signalling pathways promoting cancer development.

## The constitutive activity of US28 as a factor promoting cancer development

**Figure 2.**
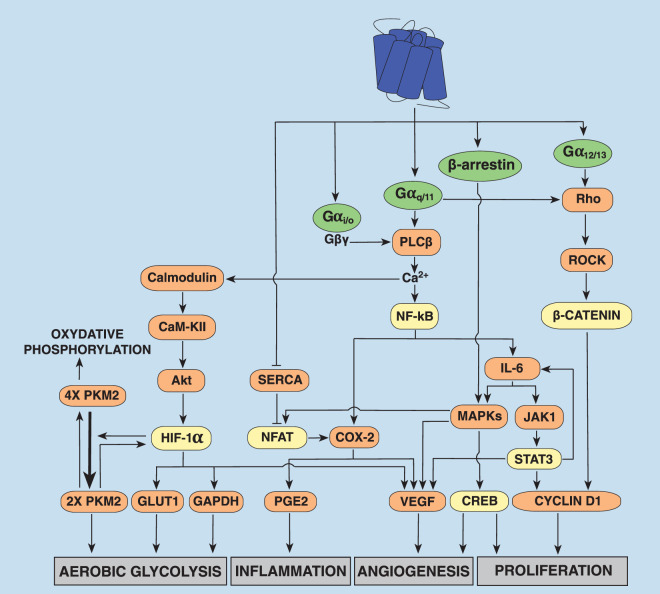
Cellular signalling pathways mediated by the constitutive activation of US28. The constitutive activation of the heterotrimeric Gα proteins from the Gαq/11, Gα12/13 and Gαi/o families as well as the constitutive recruitment of β-arrestin (in green circles) contribute to cell proliferation, angiogenesis, inflammation, and aerobic glycolysis, which are important cellular functions promoting cancer development. Each signalling pathway leading to a cell function is interconnected with the pathways controlling other functions by key multifunctional proteins. Transcription factors are depicted by yellow boxes.

Although debated, HCMV infection has been reported to have important oncomodulatory properties on numerous cancers, such as breast [45], colorectal [46–48] and prostate [49] cancers, as well as Epstein Barr virus (EBV)-negative Hodgkin lymphoma [50], alveolar soft part sarcoma [51] and gliomas [29,52–56]. Although other proteins expressed by HCMV may play a role in the oncomodulatory properties of this virus, US28-mediated constitutive G protein signalling has been associated with acceleration of cell proliferation, angiogenesis, inflammation and metabolic remodelling (Figure 2), which are important components of cancer development [57].

### Proliferation

HCMV infection modulates the host cell cycle to create an environment that is optimal for viral gene expression, DNA replication and production of infectious virus [58]. As an example, initiation of HCMV gene expression has been shown to require that the cells halt temporary in G0 or G1 at the time of infection as infection of cells in the S phase blocked immediate early gene expression [59,60]. Modulation of the cell cycle machinery is also a hallmark of cancer and sustained cell proliferation is an important part of cancer development and progression [61]. Activation of the signal transducer and activator of transcription 3 (STAT3), an oncogenic transcription factor, accelerates cellular proliferation by up-regulation of proteins involved in the control of cell cycle progression, such as cyclin D1 [62]. US28-mediated constitutive G protein activation was reported to increase the expression of cyclin D1 in mouse fibroblast NIH-3T3 cells stably transfected with US28 [24]. However, it would have been interesting to confirm these results in a human cell line. In agreement with its proliferative properties, dysregulation of STAT3 has been reported in 50–90% of all human cancers [63]. One of the receptors responsible for activation of STAT3 is the receptor of interleukin-6 (IL-6), a pleiotropic cytokine highly produced in the tumour-baring host [64]. HCMV infection has been shown to induce IL-6 secretion by infected trophoblasts extracted from term and first trimester human placentas as well as infected choriocarcinoma JEG-3 cells [65]. Targeting IL-6 signalling has also been shown to suppress survival of glioma stem cells isolated from human glioblastoma xenografts (D456MG, D54MG) or from fresh human surgical specimens and tumour growth [66]. Notably, primary glioblastoma tumours contain both US28 and activated STAT3 [27,67]. In NIH-3T3 cells stably expressing US28, HEK-293T transiently transfected with US28, and in HCMV-infected malignant glioblastoma U373MG cells, the constitutive activation of Gαq by US28 is reported to enhance cell proliferation and DNA synthesis by activating the nuclear factor kappa-light-chain-enhancer of activated B cells (NF-kB) leading to secretion of IL-6 inducing STAT3 phosphorylation via Janus kinase 1 (JAK1) [27] (Figure 2). As it has been reported that IL-6 itself is transcriptionally activated by STAT3 [68], it may lead to an IL-6-STAT3 positive feedforward loop. Recently, a novel STAT3 inhibitor, napabucasin, was shown to suppress proliferation of U87MG and LN229 glioblastoma cells [69] highlighting the important role of STAT3 activation in proliferation of these cells. Another transcription factor known to activate the transcription of cyclin D1 is β-catenin. Chronic activation of β-catenin signalling is found in a variety of human malignancies, such as melanoma, colorectal and hepatocellular carcinomas [70]. Moreover, transgenic mice expressing US28 in intestinal epithelial cells has been shown to develop adenomas and adenocarcinomas that express abnormally high levels of nuclear β-catenin protein [71]. In line with these observations, it was reported that in human foreskin fibroblasts and human glioblastoma U373MG cells infected with HCMV, the constitutive activation of Gαq and G proteins from the Gα12/13 family by US28 induces activation of β-catenin in a PLCβ-independent manner via activation of Rho and ROCK [28] (Figure 2).

### Angiogenesis and inflammation

Inflammation of host cells is a mechanism used by HCMV to increase its potential of replication. The inflammatory cytokine IL-6 has been reported to promote HCMV reactivation from latency in dendritic cells via p42/44 mitogen-activated protein kinases (MAPKs)-mediated transcriptional induction of major immediate early genes [72,73]. This has been shown to result in efficient progression of the virus life cycle and, ultimately, higher titres of infectious virus. Inflammation induced by HCMV infection may contribute to angiogenesis. HCMV infection of endothelial cells induces the expression of adhesion molecules, which enhance transendothelial cell migration of inflammatory cells including monocytes [74]. Infected monocytes are also known to disseminate HCMV throughout the body, including the vasculature [75]. The infected monocytes enter the vasculature, where they mature, initiate the viral infection programme and stimulate virus replication. Angiogenesis and inflammation are two important physiological processes promoting and supporting cancer development.

To grow, all solid tissues require a proper vasculature that provides oxygen, nutrients and waste disposal. Consequently, early activation of angiogenic processes is required to sustain proliferation of tumour cells, but also metastasis [76]. Early during tumour progression, hypoxia triggers the transcription of several genes, such as the hypoxia inducible factor-1 (HIF-1). This transcription factor contains an oxygen-regulated α subunit and a stable β subunit, which upon complex formation activates transcription of many genes involved in proliferation, angiogenesis and glycolysis [77]. HIF-1α is degraded rapidly within cells under normoxic conditions preventing dimerisation with HIF-1β to become an active transcription factor. In cancer cells, expression of oxygen-regulated subunit α is increased by either HIF-1α protein synthesis and stability or by increase in mRNA levels in normoxic conditions [77]. This occurs through mutations of regulatory proteins, but also by infection with oncogenic viruses, such as HCMV, as demonstrated in human foetal foreskin fibroblasts HFFF2 infected with HCMV (AD169 strain) 24 h post-infection [78]. HIF-1 promotes transcription of the vascular endothelial growth factor (VEGF), a key player in tumour angiogenesis [79]. US28-expressing NIH-3T3 fibroblasts and U251 glioma cells have been shown to promote the transcriptional activity of HIF-1α under normoxic conditions via constitutive activation of Gα_q_ by stimulating the calcium/calmodulin-dependent protein kinase type II (CaMKII) and the subsequent stimulation of Akt [29] (Figure 2). NF-kB constitutively activated by US28 also converges toward VEGF secretion by several pathways. One of them is by increased expression of cyclooxygenase-2 (COX-2), a key mediator of inflammation by its production of prostaglandin E2 (PGE2) [80] and a major determinant in several forms of cancer, as demonstrated in NIH-3T3 stably expressing US28 and in HCMV-infected human foreskin fibroblast BJ cells [26] (Figure 2). Hence, treatment with Celecoxib, a COX-2 selective inhibitor, has been shown to significantly reduce tumour formation in a murine model of mice injected with US28-transfected NIH-3T3 cells by repressing the US28-induced angiogenic activity. STAT3 activation by US28-mediated IL-6 secretion was also reported to be involved in VEGF production in NIH-3T3 cells stably transfected with US28 and U373MG cells 48 h after infection with HCMV as well as in primary glioblastoma specimens from patient samples [27] (Figure 2). Both p38- and p42/44-MAPKs were also reported to be involved in VEGF production by the constitutive activation of NF-kB by US28 in COS-7 cells transiently transfected with US28 [24] (Figure 2). This would need to be confirmed in the context of a HCMV infection. As the IL-6 receptor is reported to activate both p38- and p42/44-MAPK pathways [81,82], this MAPK-mediated VEGF secretion may come from the NF-kB-induced IL-6 secretion activated by US28. Additionally, although this has been shown in a heterologous expression system, the constitutive recruitment of β-arrestin seems to be required for a maximal activation of the p38-MAPK pathway [16], suggesting a partial role of β-arrestin in US28-mediated p38-MAPK activation (Figure 2). US28-mediated activation of MAPKs and of PLCβ was also shown to mediate activation of the cAMP response element binding protein (CREB) in HEK-293 cells and COS-7 cells transiently transfected with US28 [31,38], a transcription factor linked to a broad range of cellular processes, such as cell proliferation, differentiation, survival, angiogenesis, immune response, migration and invasion [83] (Figure 2). Although this has not been proven in the context of HCMV infection, in HEK-293 cells transiently transfected with US28, this activation of CREB by US28 has also been reported to stimulate the major immediate early gene promoter/enhancer (MIE) [84], thus playing a key role in the activation and replication of HCMV, but also in HCMV reactivation from latency [85].

Nuclear factor of activated T cells (NFAT) are transcription factors playing a crucial role in vascular development and are linked to transcription of VEGF [86]. Many studies also report the role of NFAT as a transcription factor that up-regulates the expression of COX-2 [87–91]. Accumulating studies have suggested that NFAT is involved in many aspects of cancer, including carcinogenesis, metastasis, cancer cell proliferation and tumour microenvironment [92]. The NFAT family is composed of five members, known as NFAT1–NFAT5. While NFAT5 is regulated by osmotic stress, NFAT1–NFAT4 proteins are activated by increased cytosolic calcium concentration [92]. Dephosphorylation of the NFAT regulatory domain results in the nuclear translocation and activation of NFAT. Different proteins constitutively activated by US28 have been shown to be involved in NFAT activation, such as PLCβ, P38- and p42/44-MAPKs, and Gi/o proteins in HEK293 cells transiently transfected with US28 [31] (Figure 2). The involvement of the constitutive activation of Gαi/o proteins has been hypothesised to be mediated through Gβγ (Figure 2). A subsequent study using the same cellular model has shown that US28 constitutively interacts with the sarco/endoplasmic reticulum calcium ATPase 2 (SERCA2) and inhibits its ATPase activity [93]. This finding would need to be confirmed in a cellular model more physiologically relevant in the context of cancer. SERCA2 transport calcium from the cytosol to the reticulum lumen against gradient via ATP hydrolysis, leading to a reduction of calcium concentration in the cytosol. US28, by constitutively inhibiting SERCA2 ATPase activity, was shown to increase cytosolic calcium concentration which favour NFAT activation and the associated increased expression of COX-2 in human umbilical vein endothelial cells (HUVECs) [93] (Figure 2). Furthermore, treatment of xenograft mice bearing sinus venosus endothelial cells (SVECs) expressing US28 with cyclosporine A, an inhibitor of calcineurin, a protein phosphatase known to dephosphorylate and consequently activate NFAT [94], was shown to reduce the induced tumour volume by 50% [93].

### Metabolic reprogramming and glycolysis

Metabolic reprogramming in host cells facilitates optimal virus production as it relies on the metabolic network of the host cell to provide energy and macromolecular precursors to fuel viral replication. In line with this affirmation, HCMV infection has been shown to enhance glucose uptake [95] and glucose withdrawal during HCMV infection to reduce virus replication [96,97]. Reprogramming of energy metabolism conferred by virus infection often mirror metabolic changes seen in cancer cells, such as up-regulation of nutrient consumption and anabolism to support rapid cell growth. Therefore, by promoting its viral replication, HCMV-mediated metabolic reprogramming also supports the high metabolic demands of cancer cells.

Transformed cells switch from the slow yet energetically favourable oxidative phosphorylation towards the fast and less glucose-efficient aerobic glycolysis [98]. Pyruvate kinase M2 (PKM2) is an important glycolytic enzyme in energy metabolism with a distinct dual role depending on its oligomeric state. Tetrameric PKM2 drives the cycle of oxidative phosphorylation [99], while the PKM2 dimer is associated with increased glycolytic flux and lactate secretion [100]. In addition, dimeric PKM2 is able to translocate to the nucleus where it functions as a co-transcription factor regulating expression of proliferative genes thereby promoting tumorigenesis [99,101,102]. PKM2 phosphorylation at tyrosine 105 has been shown to inhibit formation of the tetramer and to promote the PKM2 dimer [100,103]. PKM2 stimulates both HIF-1 activity and transcription [101,104]. Conversely, the transcription of PKM2 is under the control of HIF-1, creating a feedforward loop, enhancing activity of both key metabolic proteins [105]. In both US28-expressing NIH-3T3 fibroblasts and U251 glioma cells, an increased phosphorylation of tyrosine 105 of PKM2 and a corresponding increase of PKM2 dimer compared with mock cells was observed, and this effect was mediated by HIF-1 [29] (Figure 2). These results suggest that US28 induces metabolic reprogramming by inducing a switch from oxidative phosphorylation to aerobic glycolysis. Furthermore, it was also shown that the activation of HIF-1 enhanced by PKM2 dimer and Gαq-dependent Akt activation by US28 also increases mRNA levels of the glucose transporter type 1 (GLUT1) and metabolic enzyme glyceraldehyde-3-phosphate dehydrogenase (GAPDH) [29] (Figure 2). GLUT1 facilitates entry of glucose into cells and consequently helps to support the high rate of glucose consumption characteristic of cancer cells [106]. The catalytic function of GAPDH is one of the critical rate-limiting steps of glycolysis [107] and computational modelling and metabolomics show that aerobic glycolysis in cancer flux through GAPDH [108]. Consequently, both GLUT1 and GAPDH help to support the PKM2 dimer-mediated aerobic glycolysis.

## Conclusion

Altogether, these studies highlight the fact that the robust constitutive activity of US28 underlies a complex network of signalling pathways which may contribute to cancer development by activating four important functional axes: cell proliferation, angiogenesis, inflammation and aerobic glycolysis. Furthermore, each axis seems to be interconnected with others via involvement of multifunctional proteins able to create some ‘branching' through their respective signalling networks. These interconnection points probably contribute to the oncomodulatory role of US28 by preserving activity of all the four axes if for a given reason a protein is not functional or present in a certain cellular context. Evolution has shaped US28 to be ‘fully equipped' with several molecular tricks to modify its host to support its infection and proliferation.

## Perspectives

- **Importance of the field**: Understanding the molecular mechanisms underlying the constitutive signalling of US28 represents a promising avenue to develop new therapeutic solutions to fight against cancer. As US28 is also expressed and potently signals during the latent viral phase, the impact of investigating US28-mediated signalling can also have great impact on CMV-positive and immunocompetent population.
- **Current thinking**: Overall, there is growing evidence that US28-mediated signalling may represent one of the multifactorial aspects underlying cancer development. By tricking the host cell, this receptor initiates a multitude of signalling pathways favouring HCMV propagation and replication by activating cellular functions linked to cancer development.
- **Future direction**: Antivirals target the lytic phase and not the latent phase of the viral life cycle. As studies show that limited and almost undetectable expression of US28 during the latent phase is sufficient to robustly signal, blocking the constitutive activity of US28 by inverse agonists may be effective. Such an approach has to be actively investigated as those modulators may inhibit the US28-mediated signalling pathways that potentially contribute to cancer in HCMV-positive patients.

## Abbreviations

AC: adenylyl cyclase
AP-2: activator protein-2
ATP: adenosine triphosphate
CaMKII: calcium/calmodulin-dependent protein kinase type II
cAMP: cyclic adenosine monophosphate
COX-2: cyclooxygenase-2
CREB: cyclic AMP response element binding protein
CT: C-terminal tail
DAG: diacylglycerol
EBV: Epstein Barr virus
ER: endoplasmic reticulum
GAPDH: glyceraldehyde-3-phosphate dehydrogenase
GBM: glioblastoma multiforme
GLUT1: glucose transporter type 1
GPCR: G protein-coupled receptor
GRK: G protein-coupled receptor kinase
HCMV: human cytomegalovirus
HEK: human embryonic kidney
HIF-1α: hypoxia inducible factor 1α
HUVECs: human umbilical vein endothelial cells
ICL: intracellular loop
IL-6: interleukin-6
IP_3_: inositol trisphosphate
MAPK: mitogen-activated protein kinase
MIE: major immediate early gene promoter/enhancer
NFAT: nuclear factor of activated T cells
NF-kB: nuclear factor kappa-light-chain-enhancer of activated B cells
PAA: phosphonoacetic acid
PGE2: prostaglandin E2
PIP_2_: phosphatidylinositol-4,5-bisphosphate
PKC: protein kinase C
PKM2: pyruvate kinase M2
PLCβ: phospholipase Cβ
RhoA: Ras homolog family member A
ROCK: Rho-associated protein kinase
SERCA2: sarco/endoplasmic reticulum calcium ATPase 2
STAT3: signal transducer and activator of transcription 3
SVECs: sinus venosus endothelial cells
TM: transmembrane
VEGF: vascular endothelial growth factor
